# Oncoprotein mdig contributes to silica-induced pulmonary fibrosis by altering balance between Th17 and Treg T cells

**DOI:** 10.18632/oncotarget.2914

**Published:** 2015-02-11

**Authors:** Chitra Thakur, Michael Wolfarth, Jiaying Sun, Yadong Zhang, Yongju Lu, Lori Battelli, Dale W. Porter, Fei Chen

**Affiliations:** ^1^ Department of Pharmaceutical Sciences, Eugene Applebaum College of Pharmacy and Health Sciences, Wayne State University, Detroit, USA; ^2^ Pathology and Physiology Research Branch, National Institute for Occupational Safety and Health, Morgantown, USA; ^3^ Respiratory Medicine, The 4th Affiliated Hospital, China Medical University, Shenyang, Liaoning Province, China; ^4^ Central Laboratory, The Central Hospital of Wuhan, Tongji Medical School, Huazhong University of Science and Technology, Wuhan, China

**Keywords:** mdig, fibrosis, Th17, silica, gene knockout

## Abstract

Mineral dust-induced gene (mdig, also named Mina53) was first identified from alveolar macrophages of the coal miners with chronic lung inflammation or fibrosis, but how this gene is involved in lung diseases is poorly understood. Here we show that heterozygotic knockout of mdig (mdig+/−) ameliorates silica-induced lung fibrosis by altering the balance between Th17 cells and Treg cells. Relative to the wild type (WT) mice, infiltration of the macrophages and Th17 cells was reduced in lungs from silica-exposed mdig+/− mice. In contrast, an increased infiltration of the T regulatory (Treg) cells to the lung intestitium was observed in the mdig+/− mice treated with silica. Both the number of Th17 cells in the lung lymph nodes and the level of IL-17 in the bronchoalveolar lavage fluids were decreased in the mdig+/− mice in response to silica. Thus, these results suggest that mdig may contribute to silica-induced lung fibrosis by altering the balance between Th17 and Treg cells. Genetic deficiency of mdig impairs Th17 cell infiltration and function, but favors infiltration of the Treg cells, the immune suppressive T cells that are able to limit the inflammatory responses by repressing the Th17 cells and macrophages.

## INTRODUCTION

We had identified mdig gene in the coal miners' alveolar macrophages through differential display RT-PCR (GenBank BE441202, 2000; AY302110, 2006) [1, 2]. This gene was also discovered independently by Tsuneoka et al. in human promyelocytic leukemia HL60 cells or brain tumor T98G cells with c-myc overexpression and named myc-induced nuclear antigen 53 (mina53 or MINA) [3–6]. Mdig protein was localized predominantly in the nucleoli of cells and an alternative name, nucleolar protein 52 (NO52), was given in the literature [7]. By determining the expression levels of mdig among cancer tissues, we had shown that more than 90% of the human lung cancers exhibited increased expression of mdig relative to the non-cancerous lung tissues [2]. An increased expression of mdig had also been found in colon cancer [8], esophageal squamous cell carcinoma [9], gingival squamous cell carcinoma [10], lymphoma [11], renal cell carcinoma [12], neuroblastoma [13], gastric cancer [14], hepatocellular carcinoma [15], cholangiocarcinoma [16], and breast cancer [17], indicating a potential oncogenic role of this gene. The mdig gene encodes a 465 amino acids protein that contains a conserved JmjC domain that is commonly found in a number of histone lysine demethylases. Overexpression of mdig in lung epithelial cells or lung cancer cells resulted in a decreased level of H3K9me3 in the gene loci of rRNA, H19, and the genes in the satellite region [2, 18, 19]. In our recent *in vitro* studies, we revealed that mdig protein immunoprecipitated from mdig-overexpressing cells exhibited a moderate demethylase activity on H3K9me3 [18]. Additional studies by others suggested that mdig catalyzes histidyl hydroxylation of the ribosomal protein Rpl27a [20]. Furthermore, mdig might be an important regulator for the immune responses, especially for the T cells, as the fact that genetic disruption of the mdig gene ameliorated the allergic responses of the mice [21] and compromised the function of the T helper 17 cells (Th17) [6]. Clinically, increased mdig expression in the cancer tissues predicts poorer survival of patients with lung cancer, breast cancer and ovarian cancer [17, 18, 22].

Silica is an abundant mineral in airborne dust, particulate matter 10 (PM10), PM2.5, rock, and mineral ores. Pulmonary diseases due to silica exposure in some environmental or occupational settings, such as mining, quarrying, drilling, tunneling, abrasive blasting with quartz containing materials (sandblasting), or road construction, have been recognized for several decades [23]. The National Institute for Occupational Safety and Health (NIOSH) had estimated that about 1.7 million workers in industrial occupations and an unknown percentage of 3.7 million workers that are employed in agricultural settings are subjected to silica exposure [24]. The inhalation of large amounts of silica dust over time results in fatal, chronic, irrereversible, fibrotic or carcinogenic pulmonary diseases, such as silicosis, chronic obstructive pulmonary disease (COPD), immune disorder, and lung cancer [25, 26].

Given the fact that mdig was first identified from coal miners with chronic lung inflammation resulting from exposure to mineral dust at their work places and *in vitro* data suggested inducibility of mdig mRNA caused by silica particles [1], the present report addresses the role and mechanism of mdig in mediating silica-induced lung fibrosis *in vivo* through establishing mdig gene knockout mice. Our data indicate that heterozygotic knockout of mdig gene in mice attenuated the silica-induced fibrotic response in the lung through altering the balance between Th17 cells and T regulator cells (Treg), by impairing the infiltration and function of the Th17 cells. These data suggest that mdig may play an important role in Th17 cells that are the central regulatory immune cells during inflammation and fibrosis of the lung in response to silica or other environmental hazards or pathogens.

## RESULTS

### Establishing mdig knockout mice

**Figure 1:**
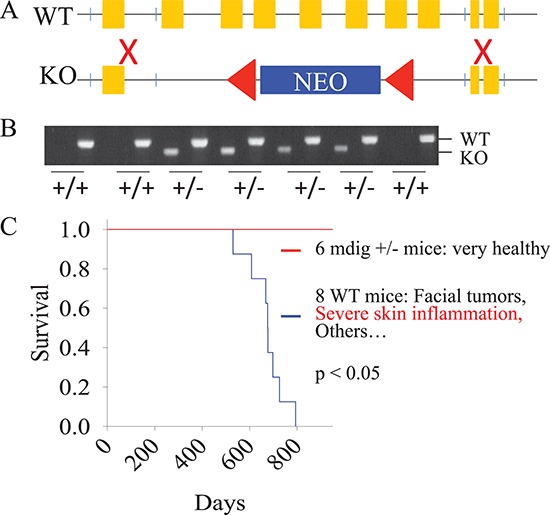
Establishment of the mdig knock-out mice **(A)** Genomic DNA from C57BL/6J mouse liver was used to amplify a 5,651 bp-longarm of mdig gene containing a 2,268 bp promoter region, the first exon (426 bp) and the 2,957 bp intron 1 region, and a 1,524 bp shortarm of mdig gene containing a 535 bp intron 8, exon 9 (90 bp), intron 9 (446 bp), and exon10 (453 bp). The longarm and shortarm of mdig gene were individually cloned into pCR-XL-TOPO vectors that were subsequently recombinated with the pPNT-targeting vector. **(B)** Genotyping was performed using DNAs from the tail tips of the F1 mice by PCR. **(C)** Kaplan-Meier survival probability analysis of the 8 WT and 6 mdig+/− mice.

To directly link mdig to lung diseases, such as pulmonary inflammation and fibrosis, in response to environmental or occupational hazards, we decided to generate mdig gene knockout mice to evaluate whether deficiency of mdig gene would reduce the burden of lung diseases induced by environmental factors. The 3′- and 5′-ends of the mdig gene were amplified using genomic DNA from C57BL/6J mouse liver followed by recombination with the pPNT-targeting vector. The region from exon 2 to exon 8 of the mdig gene was replaced by the neo cassette from the targeting vector (Figure 1A). The E14 129 ×C57 ES cells with the transfection of the recombinant vector and correct karyotype were injected into C57BL/6 blastocysts to generate chimeras and F1 mice. After further breeding for several generations, we obtained mdig heterozygotic knockout (mdig+/−) mice (Figure 1B) but not the homozygotic mice, indicating that mdig is essential for normal embryogenesis. No major phenotypic abnormality of the mdig+/− mice was found as compared to their wild type (WT) counterparts from the same progeny. In fact, the mdig+/− mice appeared to be much healthier than the WT mice during the observation period of 760 days. The WT mice, in contrast, developed facial tumors and severe skin inflammation around 550 days, suggesting that mdig may contribute to the inflammatory processes in response to microbial infection (Figure 1C). Reducing the gene dosage of mdig by heterozygotic knockout ameliorates inflammation.

### Mdig knockout attenuates silica-induced lung fibrosis

**Figure 2:**
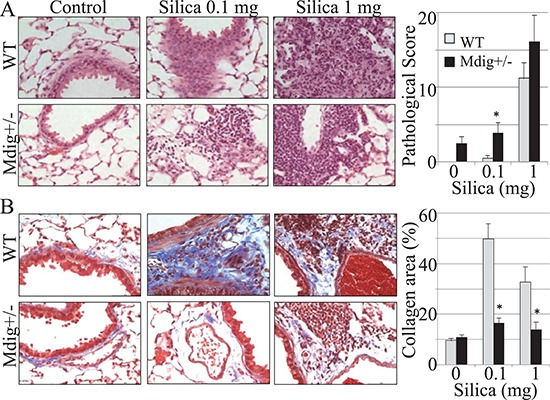
Silica induces lung inflammation and fibrosis **(A)** WT and mdig+/− mice received 2 different doses of silica (0.1 mg and 1 mg per mouse) via pharyngeal aspiration. Lung tissues were collected at 7, 28 and 84 days post silica exposure and analyzed by histology. Typical images (40×) of H&E staining of the lung tissues from the 7 days post exposure are shown. Right panel shows pathological scores of the lung tissues. Data represent the mean ± SEM of 10 mice per group. **(B)** Collagen deposition was measured by Masson's trichrome staining. Representative sections from 10 mice per treatment group per genotype at 7 days post exposure are shown. Scale bar = 50 μm. *P < 0.05 compared with respective genotypes (mdig+/− vs WT).

Since mdig was first identified from alveolar macrophages of coal miners who had history of long-term exposure to mineral dusts [1], we decided to challenge the WT and mdig+/− mice with a single treatment of silica particles (0.1 or 1 mg/mouse) through pharyngeal aspiration. At 7, 28 and 84 days post−exposure, some mice were subjected to bronchoalveolar lavage (BAL), whereas others were used for lung tissue collection and histological analysis. Pathological analyses indicated that silica induced pulmonary inflammation, such as alveolitis, lipoproteinosis, and alveolar epithelial cell hypertrophy or hyperplasia in both WT and mdig+/− mice (Figure 2A). Such inflammatory signs occurred in all groups of mice exposed to silica and appeared at all the observation time points. A high dose of silica (1 mg/mouse) amplified the pulmonary damage in both WT and mdig+/− mice. To ascertain the impact of mdig on silica-induced lung fibrosis that featured excessive collagen accumulation in the lung, we next evaluated collagen deposition in lung tissues through Masson's Trichome staining. In WT mice, silica treatment induced the formation of fibrillar collagen bundles in the periphery of granulomas and adjacent to bronchioles and blood vessels, which was persistent until 28-days post exposure (stained in blue color, Figure 2B). However, the degree of fibrosis as represented by the collagen accumulation in mdig+/− mice was significantly lower than in their WT counterparts, even in the high dose group and at 84 days post-exposure (Figure 2B). These results clearly suggest that the loss of mdig ameliorates silica-induced lung fibrosis.

### Heterozygotic knockout of mdig reduces silica-induced macrophage infiltration in the lung

**Figure 3:**
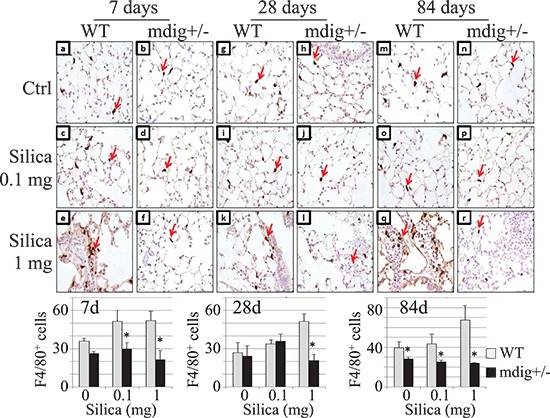
Deficiency of mdig gene reduces silica-induced macrophage infiltration into the lung **(A)** Macrophages were detected by F4/80 staining in the lungs of WT and mdig+/− mice treated with vehicle control, low (0.1 mg) or high (1 mg) dose of silica at post exposure day 7 (a–f), 28 (g–l) and 84 (m–r). Silica treatment increased the number of macrophages in the WT lung (e, k and q). In mdig+/− lungs the numbers of macrophages following silica exposure were reduced compared with the WT counterparts (f, l and r). Arrows indicate the F4/80 positive macrophage. Original magnification 40×. Scale bar = 50 μm. Representative sections from 4 mice per treatment group are shown. Bottom panels show relative quantification of the macrophages per 10 random microscopic fields (~0.1256 mm^2^). *P < 0.05 compared with respective genotypes (mdig+/− vs WT).

During inflammation, macrophages play a key role in the development of fibrosis or silicosis by releasing a number of fibrotic cytokines and inflammatory mediators. To investigate whether the attenuated collagen accumulation in the lung tissues of the mdig+/− mice treated with silica is associated with a reduced macrophage infiltration into the lung, we evaluated the numbers of macrophages on the tissue slides with the specific antibody against F4/80 antigen of the macrophages. The lower dose of silica, 0.1 mg/mouse, marginally increased the number of the interstitial macrophages at 7 days of post-exposure, but not the 28 or 84 days of post-exposure. The higher dose of silica, 1 mg/mouse, however, induced a significant increase of the macrophages in the interstitium of the WT lung at all post-exposure time points (Figure 3). In contrast, in mdig+/− mice, silica failed to induce macrophage infiltration in the lung interstitium. In fact, the macrophages were barely detected in the mdig+/− lung tissues from silica-treated animals.

### Mdig knockout increases T regulatory cells in the lung in response to silica

**Figure 4:**
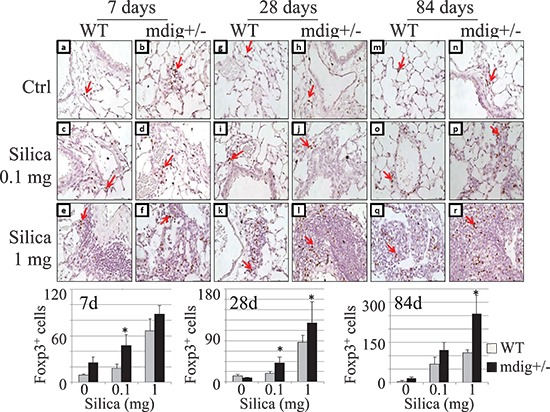
Increased Treg cells in the lung tissues of the mdig+/− mice The Treg cells were detected by Foxp3 staining in the lungs of WT and mdig+/− mice treated with vehicle control, low (0.1 mg) or high (1 mg) dose of silica at post exposure day 7 (a–f), 28 (g–l) and day 84 (m–r). Foxp3-positive cells are rarely detected in the lungs from vehicle control of WT mice in all the treatment matched groups, whereas their numbers were high in the lungs from the mdig+/− vehicle control (a-b, g-h, and m-n). Increased Treg cell infiltration was observed in silica-treated mdig+/− mice (d, j, p, f, l and r ) when compared with the WT counterparts ( c, i, o, e, k and q) in all the three different post exposure time points. Arrows indicate the Foxp3 positive Treg cells. Original magnification: 40×. Scale bar = 50 μm. Representative sections from 4 mice per treatment group are shown. Bottom panels show relative quantification of the Treg cells per microscopic field. *P < 0.05 compared with respective genotypes (mdig+/− vs WT).

The function or recruitment of macrophages is regulated by the T regulatory (Treg) T cells and T helper 17 cells (Th17). In general, Treg cells inhibit, whereas Th17 cells promote macrophage infiltration and function [27, 28]. The decreased macrophage infiltration along with the ameliorated lung fibrosis in silica-treated mdig+/− mice might be suggestive of the altered balance between Treg and Th17 cells. To explore such a possibility, we first determined the numbers of Treg cells in lung sections with an antibody recognizing Foxp3, the relatively specific transcription factor in Treg cells. Immunohistochemistry using the Treg cell marker Foxp3 revealed an increased presence of Treg cells in the lung of the mdig+/− mice in response to silica. Silica treatment with the high dose significantly increased the number of Tregs in both the WT and mdig+/− lungs at 7, 28 and 84 days post exposure (Figure 4). When compared with the WT counterparts, the numbers of Tregs were increased in the lungs from mdig+/− mice treated with both high and low dose silica. This observation indicates that loss of mdig enhances Treg infiltration into the lung during the silica-induced inflammatory response, which might account for the reduced macrophage infiltration into the lung of the mdig+/− mice exposed to silica. In other words, mdig suppresses Treg cells. Deficiency in the mdig gene, thus, may amplify the infiltration, function and proliferation of the Treg cells.

### Mdig deficiency reduces Th17 cell infiltration into the lung

**Figure 5:**
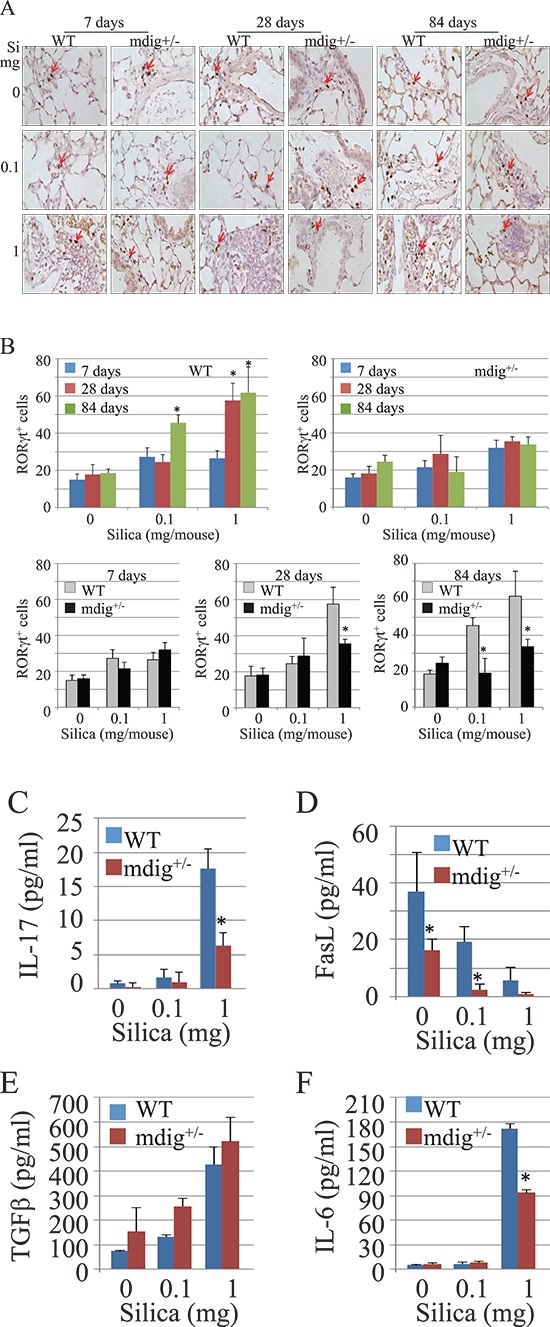
Decreased Th17 cell infiltration into the lung in mdig+/− mice **(A)** Infiltration of the Th17 cells were determined by RORγt staining in the lungs of WT and mdig+/− mice as described for the Foxp3 staining. **(B)** quantification of the RORγt^+^ Th17 cells in the lung from WT and mdig+/− mice treated with vehicle control or 1 mg/mouse of silica. **(C)** Th17 cell cytokine, IL-17A, in the BAL fluids was determined by ELISA. **(D)** ELISA assay for the levels of FasL in the BAL fluids. **(E)** ELISA assay for the levels of TGF-β in the BAL fluids. **(F)** ELISA assay for the levels of IL-6 in the BAL fluids. *P < 0.05 compared with respective genotypes (mdig+/− vs WT). Data showed are means ± SD; *n* = 4.

It has been well-established that there is a reciprocal inhibition between Treg and Th17 cells [29]. The remarkable increase of Treg cells in the lung sections from mdig+/− mice exposed to silica may suggest a certain degree of impairment of the Th17 cell function or specialization/differentiation of the Th17 cells in the mdig+/− mice. To investigate whether this is true, we next investigated Th17 cell infiltration into the lung induced by silica exposure of the WT and mdig+/− mice. We stained lung tissue sections with an antibody against RORγt, a relatively specific transcription factor of the Th17 cells. As depicted in Figure 5A, silica treatment at the dosage of 1 mg/mouse was able to increase the number of RORγt^+^ cells in both WT and mdig+/− mice at 7 days post exposure. However, the numbers of the RORγt^+^ cells were significantly decreased in the lung sections of the mdig+/− mice in comparison to WT mice at both 28 days and 84 days post exposure (Figures 5A and 5B), indicating that mdig does contribute to the function or infiltration of the Th17 cells during inflammation.

To additionally address the effect of mdig deficiency on Th17 cells, we also measured the level of IL-17A, a signature cytokine of the Th17 cells, in the BAL fluid (Figure 5C). IL-17A was barely detected in the BAL fluids from both WT and mdig+/− mice treated with vehicle control or 0.1 mg/mouse silica. The high dose of silica elevated IL-17A expression in the BAL fluids from both WT and mdig+/− mice. However, in comparison to the WT mice, a considerable reduction of IL-17A was noted in the BAL fluid from the mdig+/− mice, suggesting that the function of the Th17 cells is indeed compromised in mdig+/− mice. To additionally support this notion, we observed that the amounts of FasL, another less specific Th17 cell antigen [30], in the BAL fluids were significantly decreased in the mdig+/− mice relative to the WT mice, under either the control or silica treatment conditions (Figure 5D). A marginal increase of basal or silica-induced levels of TGFβ was noted in the BAL fluid from the mdig+/− mice (Figure 5E). There was no significant difference in the levels of IL-6 in BAL fluids from WT and mdig+/− mice treated with vehicle control or 0.1 mg/mouse silica. However, a reduced induction of IL-6 in mdig+/− mice by 1mg/mouse silica was noted (Figure 5F). TGFβ and IL-6 are the cytokines essential for specialization of the Th17 cells from naïve CD4^+^ T cells. The increase of TGFβ and the comparable level of IL-6 in the BAL fluid of the mdig+/− mice relative to the WT mice suggest that mdig knockout does not affect the maturation cytokines for the Th17 cells, but very likely impair the function of the Th17 cells, such as the release of the IL-17A or other specialization signals for the Th17 cells.

### Reduced RORγt^+^ cells in the intrapulmonary lymph nodes from mdig+/− mice

**Figure 6:**
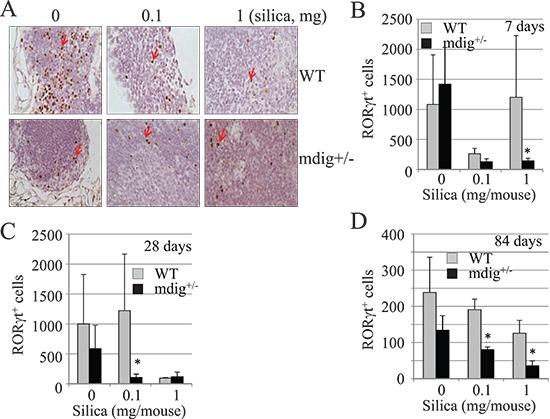
Reduced Th17 cells in the intrapulmonary lymph nodes of the mdig+/− mice **(A)** The numbers of the RORγt^+^ Th17 cells in the intrapulmonary lymph nodes of the WT and mdig+/− mice were determined by staining the slides with RORγt antibody. Data shown are lymph nodes from WT and mdig+/− mice at day 28 of post exposure. **(B–D)** Quantification of the RORγt^+^ Th17 cells in the intrapulmonary lymph nodes from WT and mdig+/− mice at 7, 28 and 84 days post silica exposure. *P < 0.05 compared with respective genotypes (mdig+/− vs WT).

To explore whether heterozygotic knockout of mdig gene affects the lineage development or specialization of the Th17 cells, we also estimated the number of the RORγt^+^ Th17 cells in the intrapulmonary lymph nodes from either the outer or hilar region of the lung. Although there are some variations in the abundance of the RORγt^+^ Th17 cells in the lymph nodes from the same group of mice, in general, the lymph nodes from mdig+/− mice exhibited fewer Th17 cells than the WT mice following silica exposure (Figures 6A, 6B, 6C, and 6D). Notably, silica treatment reduced the number of the Th17 cells in the lymph nodes from both the WT and mdig+/− mice, possibly due to migration of the Th17 cells from lymph node to the lung in response to silica exposure (Figures 6C and 6D).

### Deletion of mdig genes impairs TGFβ signaling in the immune system

**Figure 7:**
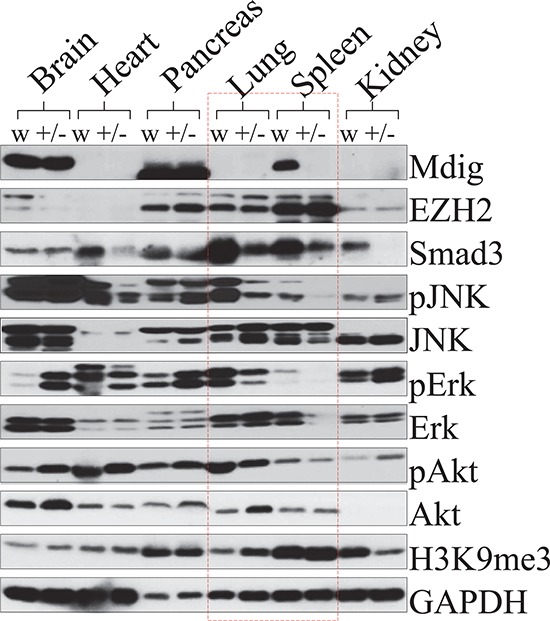
Deficiency of the mdig gene impairs smad3 expression and kinase activation in the lung and spleen Proteins from brain, heart, pancreas, lung, spleen, and kidney, were subjected to immunoblotting using the specific antibodies as indicated. All samples were obtained and analyzed in duplicate. Protein lysate of brain, heart, pancreas, lung, spleen and kidney from WT and mdig+/− mice were loaded in the same gel so that the intensity of the bands for each individual protein may be compared, which provides information on the relative abundance of each protein in the different tissues from the respective genotypes. GAPDH was used as a loading control.

The mdig+/− mice showed reduced Th17 cells in the lymph nodes as well as decreased infiltration of the Th17 cells into the lung in response to silica (Figures. 5 and 6). However, mdig+/− and WT mice expressed comparable levels of TGFβ (Figure 5E), the central cytokine that coordinates with IL-6 for specialization of the Th17 cells from the naïve CD4 T cells, indicating that deficiency in the mdig gene has less effect on the up-stream signaling for the differentiation of the CD4^+^ cells to the Th17 cells. To determine the possible mechanisms of mdig on the maturation or function of the Th17 cells, we measured systematic expression of the mdig protein in WT and mdig+/− mice. Mdig protein was undetectable in the heart, lung and kidney in both genotypes of mice (Figure 7). In contrast, tissues from brain and pancreas expressed similar levels of the mdig protein in the WT and mdig+/− mice. The undetectable expression of mdig protein in the normal lung is in agreement with our previous report showing extremely lower expression of the mdig mRNA in normal human lung tissues [2]. Considering the fact that spleen is the most important immune organ in the adult for immune cell development, special attention was paid to the expression of mdig and a number of key signaling proteins in the spleen. The mdig protein is abundant in the spleen of the WT mice. In contrast, mdig was nearly undetectable in the spleen of the mdig+/− mice (Figure 7). Correlating with this, the expression of smad3 as well as the activation of the JNK, Erk and Akt kinases were significantly reduced in both lung and spleen of the mdig+/− mice relative to the WT mice (Figure 7). Smad 3 is the pivotal receptor smad protein for TGFβ signaling. Decreased expression of smad3 in the spleen will consequently impair the TGFβ signaling required for the specialization of the Th17 cells. Similarly, reduced activation of the JNK, Erk and Akt kinases will impact the Th17 cells function negatively.

## DISCUSSION

Lung inflammation and fibrosis are the most common pathological changes resulting from inhalation of airborne particles from either environmental or occupational sources. In both patients with and animal models of fibrosis, infiltration of T cells into the lung has been frequently noted [31]. How these infiltrated T cells regulate the resolution of inflammation, repairing of the damaged tissues, and accumulation of the extracellular matrix, especially, the collagen however, remains unexplained. In the present study, we provide evidence showing that heterozygotic depletion of the mdig gene in mice ameliorates silica-induced lung fibrosis through reducing infiltration of the Th17 cells into the lung. This notion was additionally supported by the reduced levels of IL-17A in the BAL fluids from the mdig+/− mice treated with silica and the attenuated expression of the smad3, a pivotal protein in mediating the TGFβ signaling, as well as impaired activation of JNK, Erk and Akt kinases, in the spleen of the mdig+/− mice. Thus, our data suggest that mdig is able to regulate the balance of the Th17 cells and Treg cells by favoring the function or specialization of the Th17 cells.

Excess accumulation of collagen is a hallmark of pulmonary fibrosis, which is indicative of injury and remodeling process [32]. A significant increase in collagen deposition following silica exposure was observed in WT mice after 1 week post exposure. However, changes in collagen accumulation following silica exposure were not observed in the mdig+/− lungs compared to the vehicle controls at the subsequent time points (Figure 2). This observation indicates clearly that silica-induced injury and remodeling occur more rapidly in WT mice that exhibit signs of classical fibrosis (silicosis), whereas this fibrotic response is attenuated in the mdig+/− mice. It has been well-established that macrophages and T cells are important components involved in orchestrating the tissue injury and fibrotic responses in response to silica and other particulate matters through the release of fibrotic cytokines and inflammatory mediators. There was a significant reduction in the number of the infiltrated macrophages in the lung of mdig+/− mice at all post exposure times compared to their WT counterparts (Figure 3), although silicotic granulomas were observed in both WT and mdig+/− mice. This observation indicates that mdig may be involved in the migration or maturation of the macrophages in lung. As one of the innate and adaptive immune response cells, the macrophage is a regulatory target of other immune cells, such as Th17 cells and Treg cells. It has become evident that Th17 cells are the master regulators for the recruitment of the macrophages to inflammatory sites following infection or other types of injuries [27]. In contrast, Treg cells can directly suppress macrophages, especially the M1 pro-inflammatory macrophages, through inhibiting the Toll-like receptor 2 (TLR2) and TLR4 signaling pathways, leading to decreased expression of the inflammatory cytokines and/or other mediators in macrophages [33]. It has also been reported that Treg cells are capable of expressing PPARγ that induces conversion of the pro-inflammatory M1 macrophages into the anti-inflammatory M2 macrophages [34]. Although analyses of the total immune cells in bronchoalveolar lavage fluids and lymph nodes indicated comparable numbers of the total T cells between the mdig+/− mice and WT mice (data not shown), immunohistochemistry staining for the Treg and Th17 cells in the lung tissues revealed an altered balance between Treg and Th17 in the mdig+/− mice. Deficiency of the mdig gene promotes Treg cells but inhibits Th17 infiltration into the lung in response to silica challenge.

The Th17 cells are characterized by the signature cytokine IL-17A, the relatively specific transcription factor RORγt, and the requirement of the cytokine TGFβ and IL-6 for their specialization or lineage commitment [35]. Our evaluation of the Th17 signature cytokine, IL-17A, and the Th17 cell specialization cytokines TGFβ and IL-6 in the BAL fluids by ELISA revealed a decrease in the level of IL-17A, but not TGFβ, in the mdig+/− mice, suggesting that mdig may function at the down-stream of the Th17 cell specialization signals. The possible contributions of the Th17 cells to fibrotic lung diseases, such as silicosis, cystic fibrosis or hypersensitivity pneumonitis, had been reported previously [36–40]. However, in a study using IL-17 receptor deficient mice (IL-17R^−/−^), Lo Re et al. [36] showed that deficiency of IL-17R attenuated silica-induced acute lung inflammation, but had less effect on silica-induced lung fibrosis. This observation suggested that for the development of lung fibrosis, a plethora of other Th17 cell cytokines may be needed. The observed impairment of the Th17 cell infiltration and amelioration of the silica-induced lung fibrosis in the mdig+/− mice provides evidence to support this and other earlier observations.

As an originally identified mineral dust-induced gene in the alveolar macrophages from coal miners who were exposed to occupational dusts in their working places, mdig had been shown to be able to promote cell growth and cell cycle transition of some tumor cell lines [1, 2, 19]. The regulatory role of the c-myc oncogene as well as the JNK-STAT3-Akt signaling pathway on the expression of mdig further linked this gene to the uncontrolled proliferation of the cancer cells [3, 22]. In addition to its role in cell growth or proliferation, mdig had also been suggested to be an important regulator for the immune system, such as the T lymphocytes. The first report linking mdig to T cells is from studying Th2 bias in susceptible and resistant mouse strains to *Leishmania major* infection which revealed that mdig represses expression of IL-4, a Th2 cytokine [5]. A recent study using silicon nanowire technology to deliver siRNA screening library to the T cells suggested that mdig is more likely an essential regulator for the Th17 cells by promoting transcription of the Th17 signature cytokines and transcription factors [6]. Furthermore, silencing mdig by siRNA amplified expression of Foxp3, the master transcription factor of the Treg cells [6, 28]. Our *in vivo* data validated these notions, which indicated that deficiency in the mdig gene in the mdig+/− mice reduced the number of lung-infiltrating Th17 cells and the total number of the Th17 cells in lymph nodes, whereas the infiltration of the Treg cells was increased in the mdig+/− mice.

The mdig gene encodes a protein containing a conserved JmjC domain that serves as a catalytic domain for a number of histone demethylases. It remains to be clarified whether mdig protein is one of the histone demethylases. In overexpression or gene silencing experiments using immortalized or cancer cell lines, mdig exhibited some marginal activities in reducing the level of lysine 9 trimethylation of the histone H3 protein (H3K9me3) [18]. By incubating the H3K9me3-containing peptide with the immunoprecipitated mdig protein from A549 cells overexpressing the exogenous mdig, a moderate demethylase activity of the mdig protein was observed, which may be associated with the increased expression of H19, c-myc, histone demethylase jhdm3a, and the genes in the satellite region [18]. However, the recombinant mdig protein from bacteria presented histidine hydroxylase activity but not demethylase activity [20]. Moreover, the most recent structural-functional analysis revealed that the lack of the demethylase activity of mdig is largely due to the presence of the c-terminal dimerization and the winged helix (WH) domains that may prevent access of the methylated lysine to the catalytic core [41]. The discrepancy on the demethylase activity of mdig protein may be attributed to the differences in post-translational modifications and protein-protein interaction between recombinant protein and the intracellular native protein. Indeed, our recent proteomic analyses and co-immunoprecipitations suggested that mdig can form complexes with RbAp48, Ku70, TDRD3, and several other chromatin or DNA binding proteins in the cells (Wang et al, unpublished). Certainly, such interactions may possibly rearrange the overall structure of the protein, leading to changes in the demethylase activity. An additional possibility to be considered is the multiple alternatively spliced isoforms of the mdig mRNA as we had originally reported [1]. Some of these alternatively spliced products may have different structural-functional profiles.

The gene knockout experiments in this report yielded heterozygotic mdig knockout (mdig+/−) mice only but not the homozygotic knockout (mdig−/−) mice, suggesting that homozygotic deletion of the mdig gene is lethal for embryogenesis. However, the report by Mori et al. [21] indicates that the mina53−/− (mdig−/−) mice are able to reach adulthood and are fertile. This discrepancy is very likely due to the use of different recombinant strategies in disrupting the mdig gene. We replaced the entire region from exon 2 to exon 8 of the mdig gene with the neo cassette to ensure a complete deletion of the mdig gene as well as the major alternatively spliced isoforms. In studies by Mori et al, only exon 2 was replaced. Accordingly, some alternatively-spliced mina53/mdig mRNAs may exist to support embryogenesis. Nevertheless, both Mori's and our studies showed a similar effect of mdig/mina53 on the immune system. It is possible that the attenuated allergic response in mina53−/− (mdig−/−) mice is a result of the impaired function of the Th17 cells, even though Th17 cells or Th17 cell cytokines were not discussed in Mori's report [21].

In summary, we created mdig heterozygotic knockout mice that exhibited normal development and fertility. Histological assays revealed that deficiency of the mdig gene ameliorated silica-induced lung fibrosis, and reduced infiltration of the macrophages and Th17 cells into the lung interstitium in response to silica. Additional biochemical studies demonstrated that mdig may contribute to the specialization and function of the Th17 cells. In other words, the presence of the mdig gene favors the formation of lung fibrosis induced by silica through promoting the Th17 cells, the most important T lymphocytes that drive inflammation and fibrosis. These results may be clinically relevant for designing efficient treatment strategies against pulmonary fibrogenesis or other allergic diseases by targeting mdig and the Th17 T cells.

## MATERIALS AND METHODS

### Generation of heterozygous mdig knockout mice

Genomic DNA from C57BL/6J mouse liver was used to amplify a 5,651 bp long-arm of mdig gene containing a 2,268 bp promoter region, the first exon (426bp) and the 2,957bp intron 1 region, and a 1,524 bp short-arm of mdig gene containing a 535 bp intron 8, exon 9 (90bp), intron 9 (446bp), and exon10 (453bp) (Figure 1 A). The long-arm and short-arm of mdig gene were individually cloned into pCR-XL-TOPO vectors that were subsequently recombinated with the pPNT-targeting vector. The correct pPNT-long- and short-arm vectors were identified by Nhe I digestion and transfected into 129xC57 hybrid embryo stem (ES) cells by electroporation. The ES cell transfection was performed by inGenious Targeting Laboratory, Inc. in Stony Brook, NY. The positive ES clones were identified by both Southern blotting and PCR, followed by clone expansion. The successful genomic recombination in the expanded ES clone was further validated by PCR amplification using primers encompassing the integrated longarm (7,040 bp) and shortarm (1,776 bp), followed by HindIII and EcoR1 digestion, respectively. As expected, digestion of the long-arm fragment corresponding to the knockout allele (ko allele) by HindIII generated two fragments with size of 5,313 bp and 1,727 bp, respectively. Digestion of the ko allele short-arm with EcoRI produced a 1,197 bp fragment and a 579 bp fragment. Finally, the positive ES cells were microinjected into blastocysts for breeding and generating chimeras under the assistance of inGenious Targeting Laboratory, Inc.

### Pharyngeal aspiration of silica

The silica used in this study was Min-U-Sil 5 (U.S. Silica, Berkeley Springs, WV). As previously reported [42], bulk silica was examined by proton-induced x-ray emission (PIXE) spectrometry for inorganic contaminants and for desorbable organic carbon compounds by gas chromatography mass spectroscopy. These analyses determined that the bulk silica was ≥ 98.5% pure quartz with low inorganic contamination (≤ 0.10%) and only trace amounts of desorbable organic carbon compounds were found. Suspensions of silica were prepared in normal saline [0.9% (w/v) NaCl]. All animals used in this study were housed at NIOSH Animal Quarters, which is an AAALAC-accredited, specific pathogen-free, environmentally controlled facility. All procedures involving animals were approved by the NIOSH Institutional Animal Care and Use committee. Mice were anesthetized with isoflurane (Abbott Laboratories, North Chicago, IL). When fully anesthetized, the mouse was positioned with its back against a slant board and suspended by the incisor teeth using a rubber band. The mouth was opened, and the tongue gently pulled aside from the oral cavity. A 50 μl aliquot of vehicle control, 0.1 or 1 mg of silica, was pipetted at the base of the tongue, and the tongue was restrained until at least 2 deep breaths were completed. Following release of the tongue, the mouse was gently lifted off the board, placed on its left side, and monitored for recovery from anesthesia.

### Bronchoalveolar lavage and ELISA

At 7, 28 and 84 days post-exposure, mice were euthanized with an i.p. injection of sodium pentobarbital (> 100 mg/kg) followed by transection of the abdominal aorta for exsanguination. A tracheal cannula was inserted and bronchoalveolar lavage (BAL) was performed through the cannula using ice cold Ca^2+^- and Mg^2+^-free phosphate buffered saline, pH 7.4, supplemented with 5.5 mM D-glucose (PBS). The first lavage (0.6 ml) was kept separate from the rest of the lavage fluid. Subsequent lavages, each with 1 ml of PBS, were performed until a total of 4 ml of lavage fluid was collected. BAL cells were isolated by centrifugation (650 × g, 5 minutes, 4°C). An aliquot of the acellular supernatant from the first BAL (BAL fluid) was decanted and frozen for late analysis of proinflammatory cytokine levels including IL-6, TNF-α, TGF-β, and Fas Ligand with murine cytokine-specific Quantikine ELISA kits (R&D Systems, Minneapolis, MN). All the measurements were performed according to the manufacturer's instructions.

### Histopathology and immunohistochemistry

Mice used for histopathology were not lavaged. The lungs were rapidly removed from the euthanized mice and fixed by intratracheal perfusion with 1 ml of 10% neutral buffered formalin. Lungs were trimmed the same day, processed overnight in a tissue processor, and embedded in paraffin. The left lung lobe was stained with hematoxylin and eosin for routine morphologic assessment and with Masson's Trichome for evaluating fibrosis. For immunohistochemistry analyses, the paraffin-embedded lung sections were deparaffinized with xylene and hydrated in series of alcohol. To quench the endogenous peroxidase activity, slides were incubated with 1.5% to 3% H_2_O_2_ in PBS for 20 min at room temperature. Heat-mediated antigen retrieval was performed by boiling the tissue sections in citrate buffer, pH 6 for 20 min in a microwave. To block the non-specific binding of immunoglobulin, slides were incubated with a solution consisting 5% rabbit or goat serum, 0.2% triton-X 100 in PBS for 2 h at room temperature, followed by incubation with antibodies against F4/80 (1:50), Foxp3 (1:200), or RORγt (1:200) overnight at 4°C. The next day rabbit anti-rat or goat anti-mouse biotinylated secondary antibody was applied at 1:200 dilution and incubated for 2 hours at room temperature. The slides were then incubated with an ABC reagent (Vectastatin Elite ABC kit) for 45 min at room temperature and the chromogen was developed with diaminobenzidine (DAB). The slides were counterstained with haematoxylin and mounted with entellan. Omission of the primary antibodies was used as a negative control in one slide from each staining series. Collagen deposition was determined by staining the slides with Masson's Trichome. All incubation steps were carried out in a humidified chamber and all washing steps were performed with PBS. Quantification of staining was performed using Image J software (NIH) with cell counter parameters. Images were captured at 40 X, and 10 random fields from each slide were chosen in a blinded fashion from each animal under investigation All images were captured under the bright field optics of the Nikon Eclipse Ti-S Inverted microscope (Mager Scientific, Dexter, MI,) and analyzed using the Nikon's NIS Elements BR 3.2 software.

### Protein extraction and western-blotting

In some experiments, proteins were isolated from the paraffin-embedded tissue blocks using the protocol described with slight modifications. Formalin fixed paraffin-embedded lung tissue blocks were sectioned (10 μm each) and approximately 10 to 20 sections were pooled together in an Eppendorf tube, followed by addition of 1 ml octane (Sigma-Aldrich, St. Louis, MO) for deparaffinization and vortexed for 10 sec. Subsequently 100 μl of methanol was added, vortexed again and centrifuged for 15 min at 15,000 rpm. The upper layer of octane and methanol was removed and the lower residual pellet was dried under a hood for 2 to 3 min. 100 to 150 μl of 20 mM Tris-HCl buffer (pH 9) containing 2% SDS was then added to the de-waxed tissue sections, followed by heating at 100°C on a heat block for 20 min and then incubation at 60°C for 2 h. Samples were briefly centrifuged and 2 μl of the protein was used to determine the concentration using the BCA method and finally utilized for SDS-page analysis. The separated proteins were transferred onto PVDF membranes (Invitrogen, Grand Island, NY) and subjected to immunoblotting with the indicated antibodies. Primary antibodies, including mdig (mina53), smad3, phospho-smad3, TGF-β, H3K9Me3, Fas Ligand, EZH2, c-Myc, Akt, phospho-Akt, JNK, phospho-JNK, ERK, phospho-ERK, and GAPDH, were purchased from Cell Signalling (Danvers, MA), Abcam (Cambridge, MA) or Santa Cruz Biotechnology (Santa Cruz, CA).

### Statistical analysis

All numerical results are expressed as mean ± SEM. Data were analyzed by Student's *t-*test. When appropriate, the Mann-Whitney Rank Sum test was performed. Differences with a *P* value of ≤ 0.05 were considered significant.
